# Study protocol on Alzheimer’s disease and related disorders: focus on clinical and imaging predictive markers in co-existing lesions

**DOI:** 10.1186/s12877-018-0949-2

**Published:** 2018-11-14

**Authors:** Nawele Boublay, Denis Fédérico, Alain Pesce, Marc Verny, Frédéric Blanc, Marc Paccalin, Thomas Desmidt, Pierre Grosmaître, Olivier Moreaud, Solveig Relland, Estelle Bravant, Romain Bouet, Pierre Krolak-Salmon

**Affiliations:** 1 Clinical and Research Memory Centre of Lyon, Hospital of Charpennes, Hospices Civils de Lyon, Lyon, France; 2 INSERM, U1028; CNRS, UMR5292; Lyon Neuroscience Research Center, Brain Dynamics and Cognition Team, F-69000 Lyon, France; 3 Clinical Research Centre (Vieillissement – Cerveau - Fragilité), Hospital of Charpennes, Hospices Civils de Lyon, Lyon, France; 4 University Lyon, F-69000 Lyon, France; 5 Hospices Civils de Lyon, Pôle Information Médicale Evaluation Recherche, F-69424 Lyon, France; 6 Département de gérontologie clinique et centre mémoire, Centre Rainier III, Monaco, France; 7 Clinical and Research Memory Centre and Geriatrics department of Ile de France Sud, Hôpital Pitié-Salpêtrière, AP-HP, Paris, France; 8 University Pierre et Marie Curie et DHU FAST, UMR 8256 (CNRS), Paris, France; 9 Geriatrics day hospital. Geriatrics department, Memory Resources and Research Centre (CMRR), University Hospital of Strasbourg, Strasbourg, France; 10 Team IMIS/Neurocrypto, French National Center for Scientific Research (CNRS), ICube Laboratory and Fédération de Médecine Translationnelle de Strasbourg (FMTS), University of Strasbourg, Strasbourg, France; 11 Clinical and Research Memory Centre of Poitiers, CHU Poitiers, Poitiers, France; 12 Pôle de Gériatrie CHU Poitiers 86000 Poitiers, 3INSERM, CHU de Poitiers, Université de Poitiers, centre d’investigation clinique CIC1402, Poitiers, France; 13 Clinical and Research Memory Centre of Tours, CHRU Tours, Tours, France; 14 Clinical and Research Memory Centre of Lyon, Hospital of Dugoujon, Hospices Civils de Lyon, Lyon, France; 15 Clinical and Research Memory Centre of Grenoble Arc Alpin, Pôle de psychiatrie et neurologie, CHU de Grenoble, Laboratoire de psychologie et neurocognition, CNRS UMR 5105, Grenoble, France; 16 Hospices civils de Lyon, hôpital des Charpennes, 27 rue Gabriel Péri, 69100 Villeurbanne, France

**Keywords:** Co-lesions, Alzheimer’s disease, Lewy body dementia, Cerebrovascular disease, Imaging, Predictive markers

## Abstract

**Background:**

One of the crucial challenges for the future of therapeutic approaches to Alzheimer’s disease (AD) is to target the main pathological processes responsible for disability and dependency. However, a progressive cognitive impairment occurring after the age of 70, the main population affected by dementia, is often related to mixed lesions of neurodegenerative and vascular origins. Whereas young patients are mostly affected by pure lesions, ageing favours the occurrence of co-lesions of AD, cerebrovascular disease (CVD) and Lewy body dementia (LBD). Most of clinical studies report on functional and clinical disabilities in patients with presumed pure pathologies. But, the weight of co-morbid processes involved in the transition from an independent functional status to disability in the elderly with co-lesions still remains to be elucidated. Neuropathological examination often performed at late stages cannot answer this question at mild or moderate stages of cognitive disorders. Brain MRI, Single Photon Emission Computed Tomography (SPECT) with DaTscan®, amyloid Positron Emission Tomography (PET) and CerebroSpinal Fluid (CSF) AD biomarkers routinely help in performing the diagnosis of underlying lesions. The combination of these measures seems to be of incremental value for the diagnosis of mixed profiles of AD, CVD and LBD. The aim is to determine the clinical, neuropsychological, neuroradiological and biological features the most predictive of cognitive, behavioral and functional impairment at 2 years in patients with co-existing lesions.

**Methods:**

A multicentre and prospective cohort study with clinical, neuro-imaging and biological markers assessment will recruit 214 patients over 70 years old with a cognitive disorder of AD, cerebrovascular and Lewy body type or with coexisting lesions of two or three of these pathologies and fulfilling the diagnostic criteria for dementia at a mild to moderate stage. Patients will be followed every 6 months (clinical, neuropsychological and imaging examination and collection of cognitive, behavioural and functional impairment) for 24 months.

**Discussion:**

This study aims at identifying the best combination of markers (clinical, neuropsychological, MRI, SPECT-DaTscan®, PET and CSF) to predict disability progression in elderly patients presenting coexisting patterns.

**Trial registration:**

NCT02052947.

## Background

**Fig. 1:**
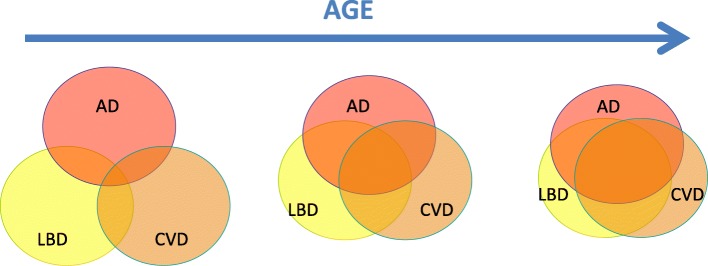
Aging and the co-occurrence of Alzheimer, vascular and Lewy types

Whereas young patients presenting a neurocognitive disorder are mostly affected by pure lesions related to Alzheimer’s Disease (AD), Vascular Dementia (VaD) that may be induced by CerebroVascular Disease (CVD) or Lewy Body Dementia (LBD), ageing favours the occurrence of co-lesions of these pathologies (Fig. 1). Age is the strongest risk factor for dementia [1]. As the populations of Western countries age, the incidence and prevalence of dementia will rise significantly. The exponential relationship between age and the prevalence of dementia, combined with the increasing number of people surviving into old age, is driving the prevalence of AD and related diseases upward, particularly LBD and CVD.

AD, the most frequent neurodegenerative aetiology of neurocognitive disorders, is marked pathologically by plaques composed of ß amyloid deposits (Aß) surrounded by dystrophic neuritis, neurofibrillary tangles composed of hyperphosphorylated tau, with activated microglia and reactive astrocytes, neuronal and synaptic loss. These lesions lead to neural loss and brain atrophy [2]. Amnestic presentation including impairment of learning and recall of recently learned information is the most common syndromic presentation of AD at the mild cognitive stage [3], latterly combined with aphasia, agnosia, apraxia and executive function impairment, some functional decline and behavioural disorders at the dementia stage [4].

LBD is marked by fluctuating cognitive decline, sleep neuropsychiatric disorders, early well-formed hallucinations, parkinsonism, and other supportive features such as dysautonomy and high sensitivity to antipsychotics [5].

CVD is the second or third most common cause of neurocognitive disorder, often associated to other pathological processes contributing to cognitive decline. Clinical symptoms depend on the location of the vascular lesions. Different CVD must be considered, i.e. multi-infarct dementia, post-stroke dementia and subcortical ischemic vascular disease [6]. Combined to clinical signs and symptoms, brain MRI is the imaging method of choice for in vivo assessment of CVD [7].

Numerous studies describe the clinical features and course of “pure” AD, in particular, the relationship between the cognitive status, behavioural disorder and the functional decline. Patients with the greatest decrease in cognitive function with respective average annual rates of decline in the Mini Mental State Examination (MMSE) show the highest decrease in score for the Instrumental Activities of Daily Living (IADL) scale as well as the most severe behavioural disturbance [8]. Far fewer data are available for VaD and AD+CVD, despite their high prevalence [9]. Patients with VaD, AD+CVD, and AD present different features at baseline and during follow-up, what underlines the need to be distinguished between them. Few studies have followed CVD or AD+CVD patients longitudinally to assess the course of their cognitive decline. Such studies have also produced conflicting results [10–12]. With regard to AD and LBD, the study of Schneider et al. had shown that a large majority of the older persons with LBD and dementia had coexisting AD pathology [13] and according to Nelson et al., the clinical diagnoses of LBD and LBD + AD were suboptimal when contrasted with autopsy results [14], which makes the assessment of cognitive status, behavioural disorder and functional decline in these underlying lesions difficult.

The aim of this study is to identify the markers, as assessed by Magnetic Resonance Imaging (MRI), Single Photon Emission Computed Tomography (SPECT-DaTscan®), Positron Emission Tomography (PET) and CerebroSpinal Fluid (CSF), combined with clinical information, that are the most predictive of functional disability progression in the elderly presenting a progressive cognitive decline related to AD, LBD, CVD and all mixed patterns. This approach will allow better defining the therapeutic targets in the elderly at a medium term.

## Methods and design

### Study aims

#### Principal objective

To identify imaging markers illustrating co-lesions of Alzheimer’s, cerebrovascular and Lewy body types of dementia the most predictive of functional disability progression.

#### Secondary objectives

1. To identify a combination of clinical and blood markers, in addition to imaging markers, the most predictive of functional disability progression,
2. To identify, in a subgroup, a combination of CSF markers, in addition to clinical/blood and imaging markers, the most predictive of functional disability progression,
3. To identify clinical/blood, imaging and CSF markers the most predictive of the cognitive decline and neuropsychiatric symptoms,
4. To evaluate the relationship between imaging and CSF markers related to co-lesions (AD, CVD and LBD) and neuropsychological performance,
5. To identify, in AD, AD+CVD and LBD patients, the links between neuro-imaging, biological and clinical markers on the one hand, and the response to AD specific treatments on the other hand.
6. To evaluate the relationship between amyloid deposition and co-lesions on the one hand, and the relationship between amyloid PET imaging markers and cognitive performances on the other.

### Study design

Multicentre and prospective cohort study with 214 patients enrolled with pathology of AD, CVD or LBD or with clinical, neuroimaging and biological markers suggesting coexisting lesions of these three pathologies and fulfilling the diagnostic criteria for dementia at a mild to moderate stage.

### Setting

The study is being conducted in 8 centres: 7 French national memory centres (Paris (AP-HP), Poitiers, Tours, Strasbourg, Grenoble, and 2 in Lyon) and 1 in the Monaco Principality.

### Characteristics of participants

#### Inclusion criteria

- Male or female subjects aged over 70 years
- Out-patient consulting at one of the Memory Centres participating in the study
- Patients meeting diagnosis criteria for dementia due to Alzheimer (McKhann, Knopman et al. 2011), cerebrovascular (NINCDS–AIREN criteria, Roma’n, G. C., Tatemichi, T. K., Erkinjuntti, T., et al. (1993), or Lewy body type (McKeith, Dickson et al. 2005), and patients presenting mixed signs and symptoms suggesting a combination of these diagnoses
- Mild or moderate dementia stage (MMSE criteria > 15)
- Being covered by health insurance
- Patients with sufficient visual, auditory and oral and written French language skills to complete the clinical and neuropsychological evaluations
- Accompanied by a close relation in sufficient contact with the subject to assess their dependency

#### Non-inclusion criteria

- Patients with psychiatric disorders (Axe 1 DSMIV disease) excepting patients with depressive or anxious disorders stabilized for more than 3 months
- Patients taking any neuroleptic psychotropic medication
- Patients taking other psychotropic medication, with the exception of any antidepressant, hypnotic, anxiolytic, acetylcholinesterase inhibitors or memantine which has been prescribed and stabilised for more than 3 months
- Patients with signs and symptoms suggestive of dementia related to diseases other than AD, CVD and LBD or mixed forms
- Patients with other neurological diseases
- Patients with progressive and unstable pathologies which could interfere with the variables under consideration
- Deafness or blindness which could compromise evaluation of the patient
- Patients being not able to undergo DaTscan®: with moderate or severe hepatic or renal impairment, a known hypersensitivity to ioflupane or any of the excipients
- If amyloid PET accepted: Patients not being able to undergo Florbetapir: with moderate or severe hepatic or renal impairment, a known hypersensitivity
- Patients living in an institution
- Patients meeting brain MRI exclusion criteria (pacemakers, aneurysm clips, artificial heart valves, ear implants, metal fragments or foreign objects in the eyes, skin, or body) or refusing MRI
- Patients being under guardianship

### Measures

All measures are reported in Table 1.

**Table 1:** Typical schedule for a patient enrolled in the CLEM protocol: summary of different stages

	Baseline	Month 6	Month 12	Month 18	Month 24
Consent	✓				
Inclusion/non inclusion criteria	✓				
Medical history or event	✓	✓	✓	✓	✓
Clinical examination	✓	✓	✓	✓	✓
CDR	✓		✓		✓
DAD	✓	✓	✓	✓	✓
IADL	✓	✓	✓	✓	✓
MMSE	✓		✓		✓
Neuropsychological diagnosis testing	✓				
ADAS-Cog/BREF	✓		✓		✓
NPI	✓	✓	✓	✓	✓
Medication	✓	✓	✓	✓	✓
Blood sampling	✓				
Neurobiotec Biobank	✓				
MRI	✓				
SPECT-DaTscan®	✓				
Amyloid PET	✓				
LP (optional)	✓				

#### Timeline

Start of recruitment: January 2014.

Duration of the recruitment period: 4.5 years.

Duration of individual participation: 2 years.

Final data collection date for primary outcome measure: July 2018.

End of the study: October 2020.

#### Outcome measures

The primary endpoint is based on the dependency progression at 2 years defined by the Disability Assessment for Dementia (DAD) scale.

#### Neuropsychological tests: Performances assessments

This assessment is included in the usual clinical practice. These tests will take place in the presence of a neuropsychologist. Besides the clinical evaluation and the initial neuropsychological inventory adapted to each team’s habits to obtain a Clinical Dementia Rating (CDR) value, all subjects will also undergo:

- ➢ For the diagnosis and correlation with imaging and CSF markers: MMSE [15] for global cognition, 16-item Free and Cued Recall Test (FCRT) to evaluate verbal episodic memory [16], Grober and Buschke [17] to evaluate visual recognition memory, Delayed Matching-to-Sample (DMS 48) [18] to evaluate visual recognition memory, DO80 [19] and Bachy-Langedock denomination task (Bachy-Langedock 1989) to evaluate language, Verbal fluency (letter P and category: animal in 2 min) [20], Working memory and executive functions with Wechsler Adult Intelligence Scale span digit test (Wechsler 1981), Trail Making test [21] and Stroop Test [22] to evaluate executive function, Visual Object and Space, Visuospatial, and visuo-perceptive abilities were studied, Perception battery [23], and Praxes and meaningless gestures comprehension test [24].
- ➢ For longitudinal assessments: MMSE, BREF [25] and Adas-Cog [26] to assess quantification of cognitive function.

#### MRI: Parameters studied and sequences

This acquisition is included in the clinical usual practice and will be performed on 3 T MRI in all centres.

A morphologic MRI will be performed as part of the battery of exploratory tests habitually carried out when a patient presents with degenerative cognitive disorders on diagnosis.

Parameters studied will be brain volume, ventricular volume, regional cortical volume, hippocampal volume, ischemic vascular lesions and microbleeds.

These analyses will perform on:

- 1 mm isotropic 3D T1 sequences without contrast injection for the quantification of Brain Parenchymal Fraction and Ventricles
- T2 gradient echo sequence, for the detection of microbleeds and amyloid angiopathy
- 1 mm isotropic 3D FLAIR sequence (only for 3 T) or 3 mm axial 2DFLAIR (1.5 T), for the quantification of vascular and bright objects
- Diffusion (b = 1000) with ADC cartography, for silent infarcts

Processing of MRI data will be performed by the “Centre d’Acquisition et de Traitement Automatisé des Images” (CATI, Head: Jean-François Mangin, Orsay). Quality control at the different steps of MRI data acquisition will be made.

#### SPECT: DaTscan® product and acquisition

Image acquisition and reconstruction will be performed with a SPECT/CT device, equipped with low-energy high-resolution (LEHR) parallel arrays, 3 h after intravenous injection of 111 to 185 MBq of 123I-FP-CIT and after oral administration of 400 mg of potassium perchlorate for thyroid uptake freezing. This exam will be conducted under the same conditions as the usual clinical practice. DaTscan (^123^I-Ioflupane Injection) is a radiopharmaceutical indicated for striatal dopamine transporter visualization using SPECT.

The acquisition parameters are standardized and will last 30 min. Quality control and processing of SPECT data will be performed by the CATI.

#### Amyloid PET: Product and acquisition

The acquisition of the PET image will start about from 30 to 50 min after intravenous injection of Florbetapir and will last 10 min. Patients must be lying on the back, head positioned so that the brain, including the cerebellum, are at centre of the field of view of the camera. It will be necessary to limit the movements of the head using adhesive strips or any flexible contention to maintain the head. The reconstruction should include a correction in order to obtain a size of pixels between 2.0 and 3.0 mm for axial images.

Florbetapir is a tracer indicated for PET imaging to estimate the density of beta amyloid plaques in the brain of adult patients with cognitive decline who are under evaluation for AD and other causes of cognitive impairment.

All data will be analysed by the CERMEP which is an in vivo multimodal dedicated to basic research and clinical imaging centre, in Lyon.

#### LP: Biomarkers

All collected samples will be sent to a central biobank for storage (Neurobiotec bank). Any use of the blood biobank will need to be approved by the study co-coordinators and the scientific committee.

The LP will follow the guidelines published in 2011 [27].

The CSF biomarkers will be determined in duplicates using standardized, commercially available ELISA Kits (Innotest β-amyloid 1–42, Tau, and Phospho-Tau (181P), Innogenetics, Ghent, Belgium). The samples will be analysed with the same batch of assay kits to decrease variability inter-lots of production of ELISA kits.

The biomarkers t-Tau, p-Tau, Aβ1–42, Aβ1–40, Total tau (t-tau),181 Phosphorylated tau (p-tau) and a-syn will be measured. If needed and depending upon the data obtained with these 3 first biomarkers, Aβ1–40 will be also measured.

### Data collection procedures

The anonymity of patients will ensure the following will be reported on the Case Report Form (CRF): the first letter of the name and surname, the number of patient inclusion corresponding to order number and the number of centre.

For each patient included in the study, case report forms anonymized includes the following informations: information to help guide the data collection, inclusion and non-inclusion Criteria to validate, patient Characteristics, clinical data, neuropsychological tests, blood sampling, MRI results, SPECT-DaTscan® results, Amyloid PET results, LP results, serious Adverse Events and withdrawal from study.

Each of these informations will exist in duplicate. Investigators will retain the duplicate, and will give the original to the Clinical Research Associate (CRA) monitor. CRF will be kept in the patient’s medical records by the investigator during the study. For each patient, the CRA will centralize slips and links and archive them. The number of inclusion will be present on each sheet of the CRF. This number of inclusion will be used to connect the various slips of the same patient. At each inclusion or withdrawal from study, the medical investigator will notify the Coordination Centre of the study by fax.

### Sample size

The Required number of subjects is estimated on the primary endpoint: disability progression at 2 years. From the following hypothesis:

- Frequency of disability progression at 2 years = 40%
- Relative risk of rapid disability progression at 2 years related to global atrophy = 1.6
- Alpha risk (α) = 5%
- Statistical power (1- β) = 90%
- Two-tailed test

The Required number of subjects is 178. With an expected drop-out rate of 20%, the number of subjects to be included is estimated at 214 patients.

### Statistic analysis

Statistical analyses will be under the responsibility of Lyon CM2R (Research and Resources Centre Memory).

#### Population descriptive analysis

Descriptive analysis of data collected from patients will be conducted. Quantitative variables will be described according to their size, mean, standard deviation, median, quartiles, and range of values. Qualitative variables will be described by numbers and percentages.

#### Principal objective: Analysis of predictive markers

An analysis of predictors of occurrence of loss of autonomy at 2 years will be conducted on patients with dementia, by providing for qualitative factors, the distribution of patients with loss of autonomy and quantitative factors, means ± standard deviation. The predictive markers will be the clinical, radiological and biological measures.

Correlations analysis will be used to compare the Pearson correlation coefficient between the DAD and markers.

Analysis of variance and covariance will be used to compare means of continuous variables, including baseline scores and changes in scores over time. In multivariate analysis based on a mixed regression model will then estimate the change in score slope of the Inclusion to 2 years and the adjusted effects of predictive markers (imaging and biomarkers).

#### Secondary objectives

The following analyses will be used to assess secondary objectives: Correlations by Pearson’s correlation coefficient, Multivariate analysis: mixed regression model, Wilcoxon signed rank test, Logistic regression models and Percentages per group will be compared by Pearson’s Chi2 test (if the numbers are expected greater than or equal to 5), otherwise by Fisher’s exact test.

#### Statistical significance level

Test results will be defined as statistically significant at *p* < 0.05.

## Discussion

The CLEM study aims at better understanding the factors underlying functional disability progression, cognitive decline and behavioural disorders in coexisting AD, CVD and LBD at mild or moderate dementia stages. Its main objective is to identify imaging markers illustrating co-lesions, the most predictive of functional disability progression.

Different combinations of biomarkers may be used in a **differential diagnosis** of neurodegenerative/vascular diseases depending on the degree of clinical relevance. First, imaging, primarily MRI, is used to eliminate a non-degenerative cause, including the different types of CVD. In this way, the International Society for Vascular Behavioral and Cognitive Disorders (VASCOG) proposed criteria for vascular cognitive disorders, in line with the DSM-5, to take into consideration the developments in other cognitive disorders such as AD [28]. Then, in order to objectify the distribution of atrophy suggestive of specific neurodegenerative disease, MRI will remain an essential tool. Thus, to distinguish AD and LBD, medial temporal lobe atrophy is mainly associated with AD diagnosis, with a good discriminatory power with LBD in pathologically confirmed cases [29]. For similar levels of dementia severity, LBD appears to have greater involvement of subcortical brain atrophy than AD [30]. Hippocampal atrophy can also be observed in VaD [31] and in frontotemporal lobar degeneration (FTLD) [32]. However, Laakso et al. found that hippocampal atrophy is significantly greater in VaD patients than in control subjects but less than in AD patients [33]. And, differently from AD, in FTLD, atrophy in the temporal lobes preferentially involves the anterior part of medial temporal lobe and the amygdala more than the hippocampus [34]. Moreover, there was evidence of a difference in trends of atrophy in the cingulate (more anterior in FTLD and more posterior in AD) [32]. Diffusion tensor MRI can also be helpful in differentiating FTLD from AD with greater damage especially in frontal white matter in FTLD [35]. However, white matter hyperintensities has been reported as similar in AD and LBD [36] while a greater number of microbleeds (MBs) was reported in LBD than in AD [37].

In addition to these brain MRI biomarkers, SPECT and PET are also of interest in the differential diagnosis between LBD and other aetiologies of dementia [38, 39]. For-example, SPECT-DatSCAN showed that presynaptic dopaminergic neurotransmission (Dopamine transporter, DAT) in substantia nigra and striatum is typically deficient in LBD whereas no deficit was observed in AD [40]. Moreover, approximately 50% of people with LBD also have amyloid accumulation visualized using amyloid PET, in a very similar distribution to that seen in AD, but to a lesser extent and with amyloid plaques more often diffuse rather than neuritic [41, 42]. Other brain imaging markers, such as 99mTc-HMPAO SPECT and FDG-PET modality also support corroborative signs of focal atrophy and can aid the differentiation of AD, FTLD, VaD, and LBD [43].

Since alterations in the CSF have been detected up to decades before the appearance of clinical symptoms in particular settings [44], the value of CSF analysis is also relevant in the differential diagnosis. In this way, CSF amyloid and tau biomarkers can for-example distinguish AD from VaD with a specificity of 80% [45], but CSF amyloid is not helpful in distinguishing between AD and LBD [46].

With increasing age biomarkers are less efficient for differential diagnosis of neurodegenerative disease, probably due to **co-existing lesions**. The etiological diagnosis is difficult when patients disclose symptoms suggestive of various diseases. Although many reviews have addressed diagnostic and therapeutic issues in pure AD, CVD and LBD, very few have focused on coexisting lesions. For example, LBD and AD are distinct disorders but they often coexist. Indeed, in LBD patients, the density of senile plaques may be similar to that observed in AD [47] and these cases are often regarded as ‘mixed’ cases of LBD with associated AD (LBD/AD). Armstrong et al. showed that the Abeta pathology of LBD/AD cases is different to that observed in patients with AD alone [48].

Regarding AD and CVD, some evidence from amyloid PET imaging suggests that increased vascular risk [49, 50] such as hypertension, diabetes, and smoking and CVD [51, 52] such as stroke, lacunar infarcts, CAA, microbleeds, and WM changes may accelerate amyloid production/aggregation/deposition and thus contribute to the pathology and symptomatology of AD [53]. One of the mechanisms linking CVD to AD is decreased cerebral blood flow, which modulates amyloid precursor protein cleavage enzymes leading to increased amyloid production [54]. Additionally, the association of the APOE4 genotype with an increased risk for both AD and CVD further suggests a potential link between CVD, and AD [55]. At the same level of cognitive impairment, AD patients with concomitant CVD were reported to be older and more severely demented, but have less severe AD pathology than patients without CVD [56]. A combination of AD and CVD is usually registered as a close third, moving up to first or second in rank in community-based studies of the oldest of old.

Since then, it seems important to establish consensus about diagnosis criteria for underlying lesions that contribute differently in the **progression of cognitive, behavioural and functional impairment** compared to pure pathology. Most of the studies report on functional, behavioural, and clinical abnormalities in patients with pure pathologies. For example, in AD subjects, autonomy loss correlates with frontal, temporal, and occipital structure atrophy [57]. In the LBD group, increased rates of cortical thinning in the frontal and parietal regions are significantly correlated with motor deterioration [58]. Other studies comparing functional ability between pure pathologies showed that VaD may be associated with a faster decline in physical functionality compared to AD [59]. Another study of 84 patients showed that LBD patients were more functionally impaired and had more motor and neuropsychiatric difficulties than patients with AD with similar cognitive scores [60].

But less is known regarding coexisting lesions in the assessment of the progression of cognitive, behavioural, and functional impairment. Yet, according to different studies, AD/LBD combination tends to induce greater cognitive and behavioural impairment than pure LBD [61, 62]. A progressive cognitive impairment occurring after the age of 70 is often related to mixed lesions of neurodegenerative and vascular origins. This complicates one of the crucial challenges for the future of therapeutic approaches in the elderly which is to target the main pathological process responsible for disability. Indeed, Schäufele et al. showed that, by controlling the degree of severity of dementia variables, only age and disability contributed to the prediction of mortality in patients with Alzheimer type, vascular or mixed dementia [63]. The longitudinal design using imaging, biological, and clinical biomarkers may give insight into helping to predict the precise role and weight of each brain lesion type in the disability progression in the co-existing pathologies and to develop preventive strategies to reduce the burden of disability related to these co-lesions.

### Limitations and strengths

The CLEM study may have some limitations. Firstly, recruitment may be difficult. Given the age of the patients, this is a dense protocol including several imaging techniques and clinical and biological assessments even if some are optional. Ideally, this study would have necessitated to follow-up the subjects for a long time but longitudinal studies of LBD for example are difficult owing to the higher mortality rates compared to AD [64].

Also, neuropathological correlates may be lacking, since discrepancies between pathological findings and clinical, as well as neuroimaging data are well known. Patient autopsy would be interesting to establish convergence evidence from imaging and biological diagnosis.

The present study also has strengths. Literature often assesses pure pathology but considers coexisting pathology only at the time of death. Moreover, to our knowledge, CLEM is the first study to follow during 2 years the disability of patients with presumed co-lesions as assessed with clinical, radiological and biological markers.

### Perspectives

It is crucial to better understand the predictors of the responses to current or future specific treatments in elderly patients affected by neurocognitive disorders. Since efficiency of disease modifying drugs has not been disclosed yet, and since side effects must be carefully considered, we aim to better define the population who would benefit from specific symptomatic or disease modifying drugs. These drugs will increasingly have to target the actual pathological processes responsible for functional decline in the medium term.

This program will allow further research on a larger cohort, in particular to validate a predictive score of functional disability risk in elderly presenting with progressive cognitive decline. A brain donor program will be developed and based on this cohort to further study the intimated neuropathological processes associated to dementia in the elderly at the terminal stage. This will also help in adapting non-drug therapeutic approaches and co-morbidity care, as well as the prevention of impairment of quality of life for the patients and their caregivers, risk of institutionalization, and costs of care.

## Abbreviations

AD: Alzheimer’s disease
ADAS-cog: Alzheimer’s Disease Assessment Scale-Cognitive Subscale
ADL: Activities of Daily Living
ANSM: Agence Nationale de Sécurité des Médicaments French Agency for the Safety of Health Products
ARWM: Age-Related White Matter Changes
BREF: Batterie Rapide d’Efficience Frontale
CAA: Cerebral Amyloid Angiopathy
CDR: Clinical Dementia Rating scale
CHU: Centre Hospitalier Universitaire
CMRR: Centre de Mémoire, de Ressources et de Recherche
CPP: Comité de Protection de Personnes (French Committee)
CRA: Clinical Research Associate
CRF: Case Report Form
CSF: Cerebro-Spinal fluid
CVD: CerebroVascular Disease
DAD: Disability Assessment for Dementia
DMS: Delayed Matched to Sample
DO: Denomination Objet
EDF: Echelle de Dysfonctionnement frontal
FCRT: Free and Cued Recall Test
FLAIR: Fluid Attenuated Inversion Recovery
IADL: Instrumental Activities of Daily Living
LBD: Lewy Body Dementia
LBD: Lewy Body Dementia
LP: Lumbar Puncture
MBs: Microbleeds
MCI: Mild Cognitive Impairment
MMSE: Mini-Mental State Examination
MRI: Magnetic Resonance Imaging
MTA: Medial Temporal Atrophy
NPI: NeuroPsychiatric Inventory
PET: Positron Emission Tomography
RL/RI 16: Rappel Libre/Rappel Indicé 16 mots
SAE: Side Adverse Event
SPECT: Single Photon Emission Computed Tomography
TMT: Trail Making Test
VaD: Vascular Dementia
VBM: Voxel Basel Morphometry
